# Aromatherapy with single essential oils can significantly improve the sleep quality of cancer patients: a meta-analysis

**DOI:** 10.1186/s12906-022-03668-0

**Published:** 2022-07-14

**Authors:** Hui Cheng, Lu Lin, Shaotong Wang, Yueyue Zhang, Tingting Liu, Yang Yuan, Qiuyun Chen, Li Tian

**Affiliations:** 1 The First Affiliated Hospital of Soochow University/School of Nursing, Soochow University, No. 188 Shizi Street, Suzhou, 215006 China; 2 School of Nursing, Medical College of Soochow University, Suzhou, 215006 People’s Republic of China

**Keywords:** Aromatherapy, Cancer patients, Sleep quality, Meta-analysis

## Abstract

**Objective:**

To investigate the effect of aromatherapy on sleep quality in cancer patients.

**Methods:**

Published literature on the effect of aromatherapy in cancer patients with sleep disorders in the form of randomized controlled trials (RCTs) were systematically retrieved and screened from PubMed, Cochrane Library, Embase, CBM, CNKI, VIP, and Wanfang databases from inception to November 2021. The methodological quality of the included studies was critically and independently evaluated by two reviewers using the Cochrane Risk of Bias Assessment Tool for RCTs. The correlated data were extracted using the pre-designed form, and all analyses were performed using Reviewer Manager version 5.4. Due to the difference in sleep quality instruments, the data extracted in this study were in the form of standard mean difference (SMD).

**Results:**

Ten RCTs included 933 patients (experimental group: 474, control group: 459), and the risk of bias in the included studies was moderate. Aromatherapy could significantly improve the sleep quality of cancer patients [SMD = − 0.79, 95% CI (− 0.93, − 0.66), *p* < 0.01], especially those with breast cancer [SMD = − 0.98, 95% CI (− 1.57, − 0.40), *p* < 0.01]. Aromatherapy with single essential oil had a better effect on sleep quality [SMD = -0.94, 95%CI (− 1.25, − 0.62), *p* < 0.01], of which lavender essential oil had the best effect [SMD = -1.06,95%CI (− 1.49, − 0.63), *p* < 0.01] while compound essential oils had no effect on sleep quality improvement in cancer patients [SMD = -0.21, 95%CI (− 0.57, 0.14), *p* = 0.23]. Four of the ten RCTs reported the occurrence of adverse events, of which only one RCT indicated that patients had headache and sneezing while the remaining six did not.

**Conclusions:**

This meta-analysis of 10 RCTs reveals that aromatherapy with single essential oil had a substantial effect on the sleep quality of cancer patients and should be recommended as a beneficial complementary therapy to promote sleep quality in cancer patients.

**Supplementary Information:**

The online version contains supplementary material available at 10.1186/s12906-022-03668-0.

## Introduction

According to the data updated to 2020, over 40 million people are affected by cancer [1], which causes many negative consequences such as sleep disorder. Among cancer-related symptoms after treatment, sleep disorders are the most common symptom among cancer survivors [2–4] and it cannot be managed effectively in post-treatment care. The incidence of sleep disorders in cancer patients is from 30% [5] to 93.1% [6]. Sleep disorders can also cause a series of chain reactions, such as depression, pain, circadian rhythm disorders, fatigue, decreased quality of life, disease progression and even lower survival rates [3, 7]. Given the importance of ensuring a good sleep, an increasing number of researchers are turning their attention to the measures to improve sleep quality.

The National Cancer Institute recommends that sleep treatment for cancer patients consists of both pharmacological and non-pharmacological treatments. Pharmacological treatments can lead to serious side effects [8], such as drug dependence, lethargy, mental confusion, etc. As a result, most guidelines support only the short-term use of medications to treat insomnia, and non-pharmacological approaches to improve sleep are attracting more attention. As a type of non-pharmacological treatment, aromatherapy has been known as one of the most commonly used complementary therapies [9], which is used all over the world because it is cost-effective, non-invasive and low in side effect [10]. Aromatherapy is a treatment that uses concentrated essential oils extracted from herbs, flowers and other plant parts to treat various diseases. As a form of herbal medicine, it has been used for thousands of years in countries such as Egypt and India [11]. Essential oils are volatile oils with different dosage forms, including inhalation type, oral type and topical type [12]. Aromatherapy makes use of the interaction of essential oils with the olfactory system to affect the connection between body and mind [11, 13], thereby improving the physical condition.

Increasing attention has been paid to aromatherapy as an alternative therapy for improving sleep in cancer patients. However, previous studies on the effect of aromatherapy on sleep quality have yielded controversial results. Therefore, the purpose of this study was to systematically review the existing studies, evaluate the effect of aromatherapy on the improvement of sleep quality in cancer patients, and provide evidence and reference for the application of aromatherapy in constructing an effective sleep regimen in cancer patients.

## Methods

This study was conducted in accordance with the Preferred Reporting Items for Systematic Reviews and Meta-Analyses (PRISMA) guidelines [14].

### Search strategies

Seven databases (Cochrane Library, PubMed, Embase, CBM, Wanfang, VIP, and CNKI) were systematically searched from inception to November 2021 for randomized controlled trials (RCTs) with no language restrictions. Two researchers independently read the title, abstract and full text to screen the studies that were eligible for the meta-analysis. If there was any dispute, a third party would be asked to make an arbitration. In addition, the reference lists of the identified articles were used to search for further potentially relevant articles. (The specific search strategy for PubMed is shown in Appendix as an example.)

### Inclusion criteria

#### Participants

Patients who were ≥ 18 years old, diagnosed with cancer and met the diagnostic criteria for sleep disorders (regardless of age, gender, ethnicity, cancer type, cancer stage and current form of treatment).

#### Intervention and control group

The intervention group used aromatherapy as the intervention measure on the basis of symptomatic treatment; the control group used usual care (including basic nursing, psychological nursing and health education and so on) or placebo care.

#### Outcomes

Trials using sleep quality as a primary or secondary outcome and containing extractable sleep quality scores were included. Sleep quality was measured using assessment tools, such as the Richards-Campbell Sleep Questionnaire (RCSQ) and the Pittsburgh Sleep Quality Index (PSQI). (The description of assessment tools is shown in Supplement Table 1).

#### Study design

Only randomized controlled trials (RCTs) were eligible.

### Data extraction

Two researchers extracted information and data independently from the included RCTs, and a third party was invited to make an arbitration in the case of any disagreement. Basic data extracted mainly included: first author and publication year of the study, characteristics of patients, characteristics of intervention (intervention form, time, frequency, dosage, program length) and control group (control measure, program length or contamination), type of aroma oils, outcome indicators and adverse events.

### Risk of bias assessment

The included studies were independently assessed by two reviewers using the Cochrane Handbook for Systematic Reviews to determine risk of bias [14]. Methodological quality was evaluated from the following seven aspects: random sequence generation, allocation concealment, blinding of participants and personnel, blinding of outcome assessment, incomplete outcome data, selective reporting, and other sources of bias. Each category was scored as low, unclear, or high risk of bias. Disagreements were resolved by discussion between the two reviewers. If no agreement was reached, arbitration by a third person resolved the issue.

### Data analysis

Review Manager version 5.4 was used for data analysis. Heterogeneity (variability of participants, interventions, and outcomes) and methodological heterogeneity (variability of study design and risk of bias) were first assessed with *I*^*2*^. If *P* > 0.1 and *I*^2^ < 50%, the fixed effect model was used. If *P* < 0.1 and *I*^*2*^ ≥ 50%, the random effects model was adopted [14]. Statistical heterogeneity was the result of clinical or methodological diversity or both in the study [14]. Subgroup analysis was performed if moderate clinical heterogeneity was found. Continuous data were synthesized using Mean difference (MD), Weighted Mean difference (WMD) and standard Mean difference (SMD). Sensitivity analysis was used to investigate the influence of fixed effects or random effects model analysis on results and any assumptions that had heterogeneity. Reporting and publication bias were assessed by visually examining the degree of asymmetry of a comparison-adjusted funnel plot made from outcome indicators.

## Results

### Literature search

**Fig. 1:**
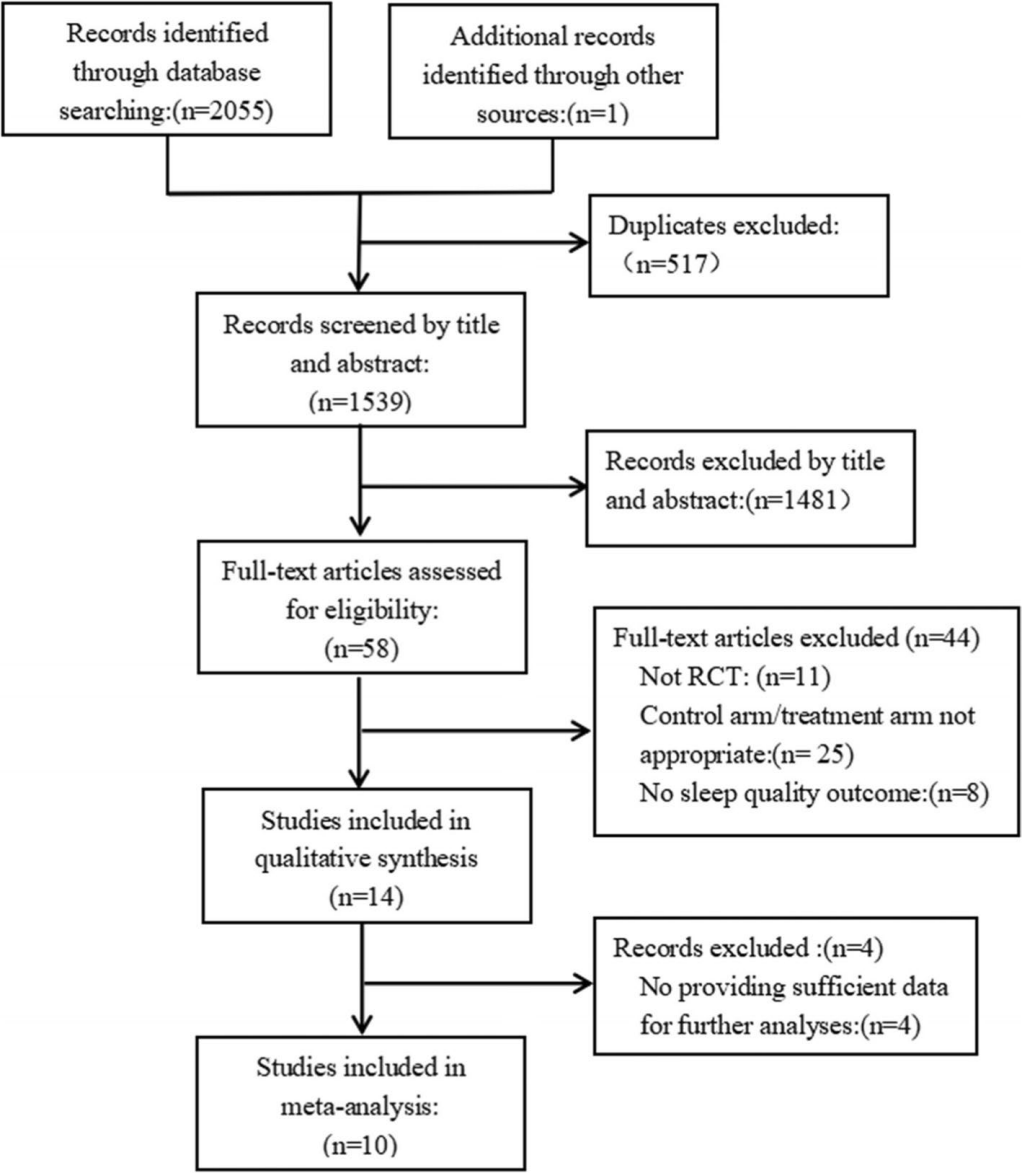
Flow chart diagram of literature identification and selection

A total of 2056 articles were retrieved, and 517 articles were deleted after duplicate checking. After repeatedly reading the title and abstract of the articles to exclude ineligible studies, 58 articles were left. When reading the full text, 44 articles were deleted (11 non-RCTs, 25 articles with inappropriate intervention or control measures, 8 articles without sleep quality outcome indicators). A further 4 articles were excluded for having not provided sufficient data for further analyses. Therefore, a total of 10 articles were included in the final meta-analysis [15–24], including 933 patients (see Table 1). The process of literature identification and selection is shown in Fig. 1.

**Table 1:** Studies included in the meta-analysis

Author, year	Sample size	Mean age(±SD) gender (male/female)	Ethnicity	Cancer, Cancer stage	Treatment status	Intervention group: form of intervention, time, frequency, drops/per time, length of intervention	Type of aroma oils	Control group: control measure, length of program, contamination	Sleep quality scale	Adverse events
Zhou et al.,2021 [15]	AG 43 CG 43	44.00 AG 30/13 CG 23/20	Chinese	liver cancer Not reported	Perioperative period	Aromatherapy inhalation 9 p.m., 1 time per day,5 drops, 2 weeks	Lavender	Usual care, 2 weeks	PSQI	Not reported
Shammas et al., 2021 [16]	AG 24 CG 17	AG 46.70 CG 48.20 female	American	Breast cancer Not reported	Perioperative period	Aromatherapy inhalation One day before surgery until discharge,4 drops	Lavender	Placebo (Coconut oil), One day before surgery until discharge	RCSQ	None
Hamzeh et al.,2020 [17]	AG1 40 AG2 40 CG 40	49.47 ± 14.52 AG1 14/26 AG2 16/24 CG 22/18	Iranian	Various Not reported	Not reported	Aromatherapy inhalation 9 p.m.,1 time per day,3 drops,1 week	AG1 Peppermint AG2 Lavender	Placebo (Aromatic distilled water), 1 week	PSQI	Not reported
Kawabata et al.,2020 [18]	AG 27 CG 30	AG 76.50 ± 10.70 11/16 CG 74.90 ± 12.90 13/17	Japanese	Various Advanced cancer	Not reported	Aromatherapy massage 8 p.m.,1 time,1 day	Compound essential oils (Lavender and orange)	Usual care, not reported	RCSQ	Not reported
Li et al.,2020 [19]	AG 52 CG 48	AG40.70 ± 6.20 CG 42.40 ± 7.30 female	Chinese	Breast cancer I–IV	Undergoing chemotherapy	Aromatherapy inhalation 8 p.m.,1 time per day,2 drops,6 mouths	Lavender	Usual care, 6 courses, not reported	PSQI	Not reported
Heydarirad et al., 2019 [20]	AG1 15 AG2 15 CG 15	AG1 47.60 ± 10.76 3/12 AG2 50.00 ± 13.94 6/9 CG 50.20 ± 18.54 8/7	Iranian	Not reported	Not reported	Aromatherapy inhalation Half an hour before going to bed,1 time per day,5 drops,2 weeks	AG1 5% rose AG2 10% rose	Usual care, 2 weeks, not reported	PSQI	Headache: 2 Sneezing: 1
Blackburn et al.,2018 [21]	AG 22 CG 28	50.00 22/28	American	Leukemia Not reported	Undergoing chemotherapy	Aromatherapy inhalation 9 p.m.,1 time per day,8 drops,3 weeks	Lavender/ Peppermint/ Chamomile	Placebo (Rose water), 3 weeks	PSQI	None
Ozkaraman et al., 2018 [22]	AG1 30 AG2 20 CG 20	AG1 57.73 ± 12.81 6/24 AG2 57.55 ± 12.87 3/17 CG 59.65 ± 13.37 2/18	Turk	Various Not reported	Undergoing chemotherapy	Aromatherapy inhalation 9 p.m.,3 drops,2 courses	AG1 Lavender AG2 Tea tree oil	Usual care, not reported	PSQI	Not reported
Li et al., 2018 [23]	AG 60 CG 60	63.20 ± 9.02 89/31	Chinese	Stomach cancer I–IV	Perioperative period	Aromatherapy inhalation 9 p.m.,1 time per day,1 day	Geranium	Usual care, not reported	PSQI	Not reported
Qi et al., 2016 [24]	AG 34 CG 35	AG 62.12 ± 10.13 21/13 CG 62.00 ± 8.83 19/16	Chinese	Colorectal cancer I–III	Perioperative period	Aromatherapy inhalation Afternoon and night,2 time per day,3 drops,10 days	Compound essential oil (Lavender, geranium, bergamot, prepared according to 1:2:3)	Usual care not reported	PSQI	Not reported

*Notes*: CG Control Group, *AG* Aromatherapy Group, *RCSQ* The Richards-Campbell Sleep Questionnaire, *PSQI* The Pittsburgh Sleep Quality Index

### Characteristics of the included trials

The characteristics of the patients, interventions, controls, outcome measures, and adverse events are shown in Table 1.

#### Participants

A total of 933 participants were included in the ten RCTs. All the participants were diagnosed with cancer. The mean age of the participants ranged from 40.70 to 76.50 years. Ten trials clearly reported ethnicity, and four trials reported cancer stage. Regarding the type of cancer, 2 trials were on breast cancer, 1 trial on liver cancer, 1 trial on colorectal cancer, 1 trial on leukemia, 1 trial on gastric cancer, and the remaining 3 on mixed cancer types. In terms of current treatment, the participants in 3 trials were undergoing chemotherapy; the participants in 4 trials were in the perioperative period; the remaining trials did not report the form or status of current treatment.

#### Interventions

All trials used aromatherapy as the intervention measure for sleep disorder. The form of intervention in nine trials was inhalation while that in one trial was massage. The frequency of treatment was 1-7 times per week; the dosage of aroma oils was 2-8 drops per time; the time of intervention in all trials was in the evening.

#### Controls

Among the ten trials included in the meta-analysis, seven trials used “usual care” [15, 18–20, 22–24], and three trials used “placebo” [16, 17, 21] as the control measure.

#### Risk of bias in individual trials

**Fig. 2:**
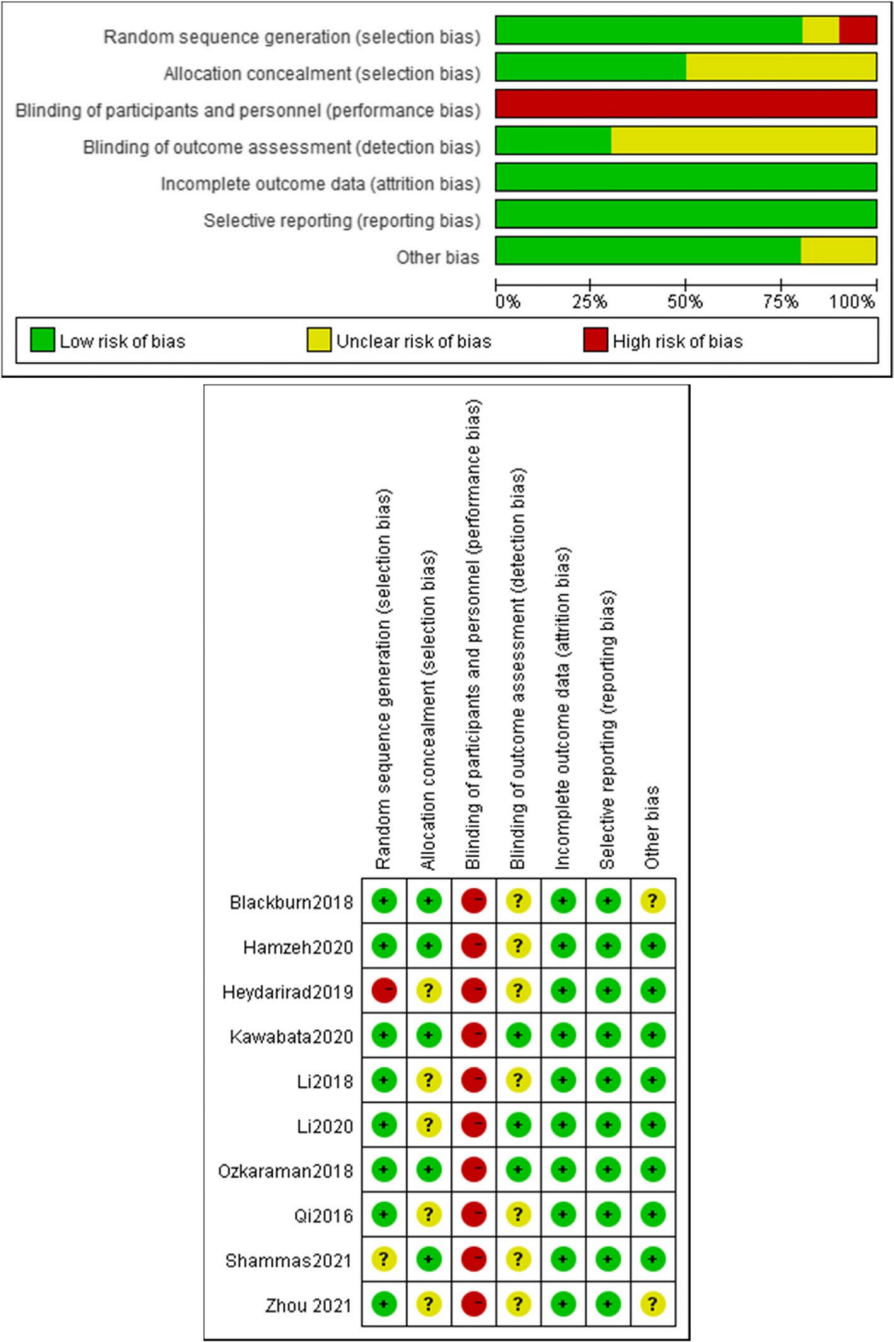
Risk of bias assessment using the Cochrane tool: (A) Overall risk of bias; (B) Risk of bias by individual trials

The methodology quality of the included trials was evaluated using the Cochrane Risk of Bias Assessment Tool for RCTs (Fig. 2). Eight studies reported the method of random sequence generation [15, 17–19, 21–24], among which one had an unclear [16], risk and one had a high risk of bias [20]. Five trials reported allocation concealment methods [16–19, 22] while five trials did not [15, 19, 20, 23, 24]. Aromatherapy interventions are difficult to blind participants and personnel; therefore, all ten trials had a high risk of bias in this domain. Three trials reported blinding of outcome assessment [18, 19, 22], while the rest had an unclear risk of bias. Only two trial was rated unclear in terms of other sources of bias [15, 21] while the other eight trials had a low risk of bias in this domain. As for selective reporting, the risk of bias was low. With regard to incomplete outcome data, the outcome data in all ten trials were relatively complete, so they were all rated low risk. Overall, the risk of bias in the included studies was moderate.

#### Analysis of overall effects

**Fig. 3:**
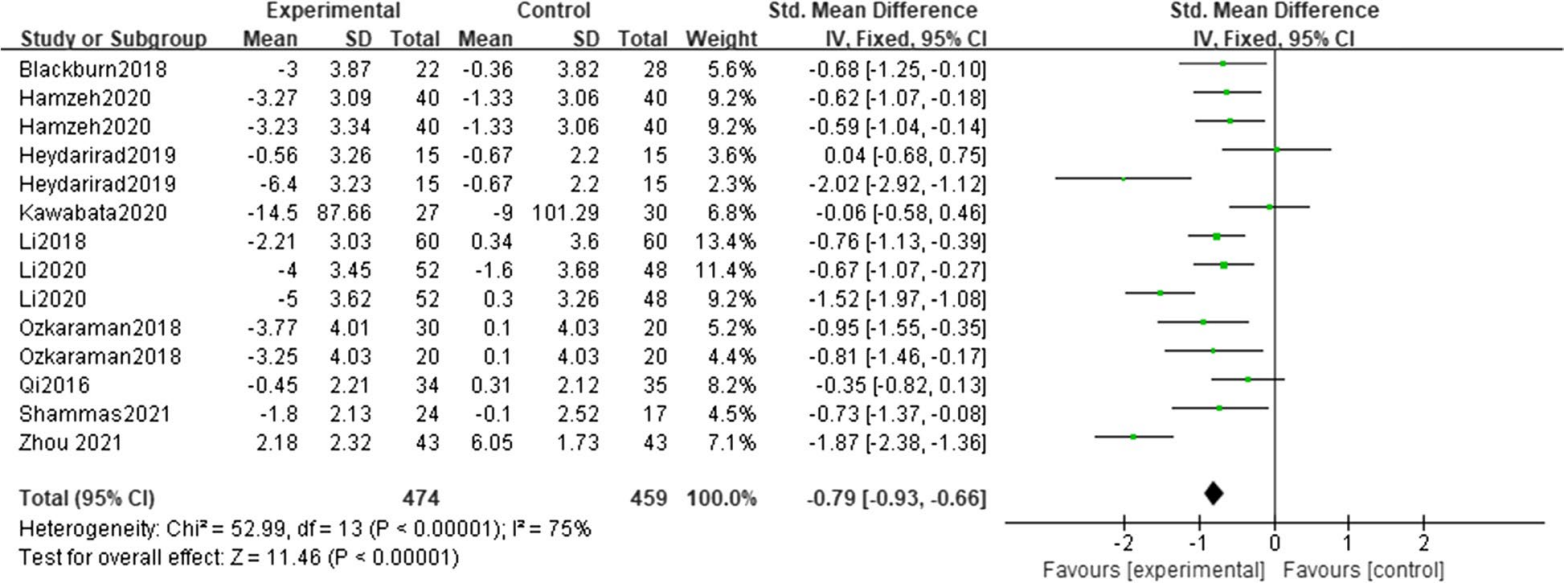
Overall effect of aromatherapy on sleep quality

**Fig. 4:**
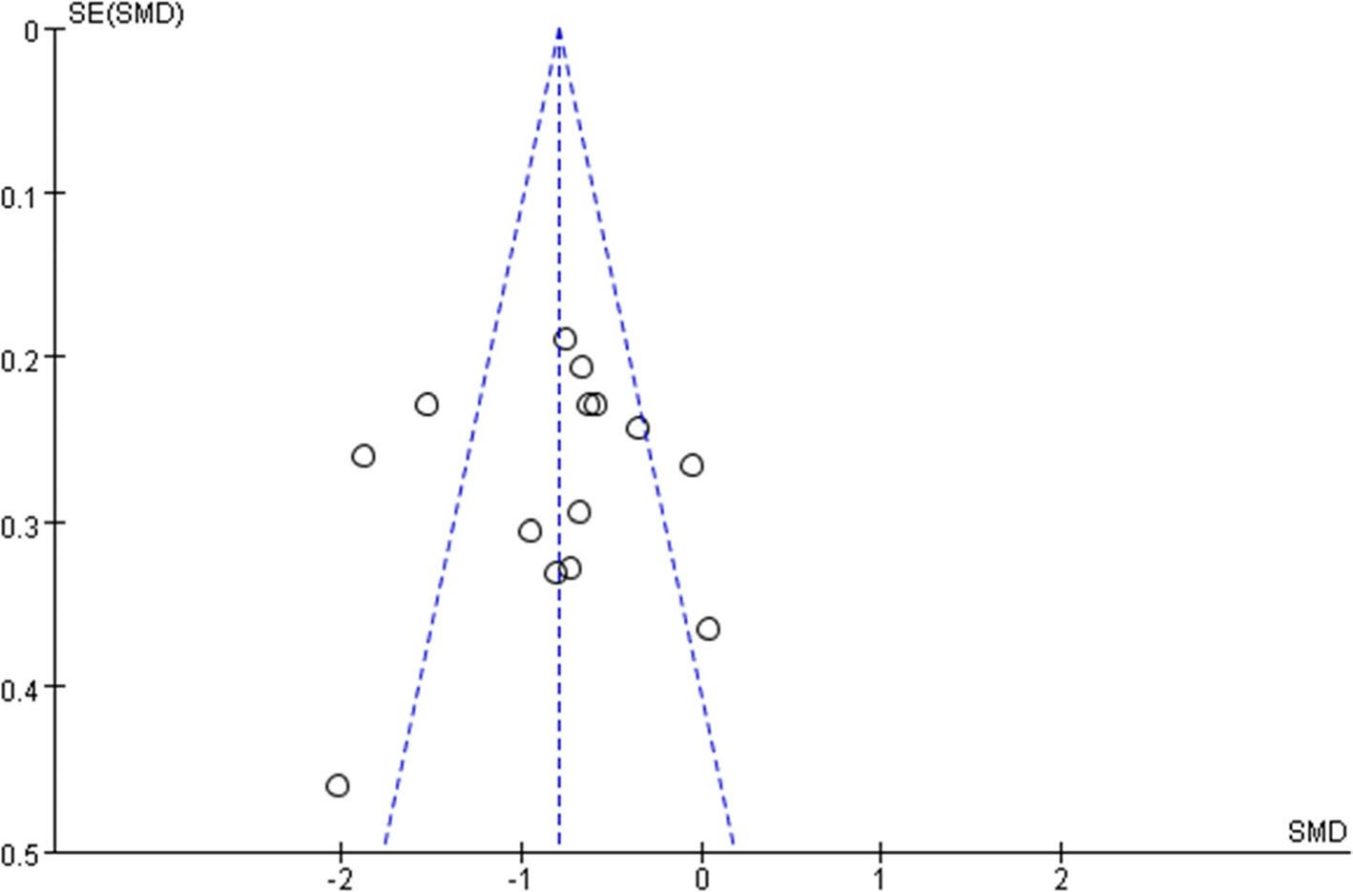
Funnel plots of sleep quality

Ten trials reported the sleep quality of cancer patients before and after intervention. The sleep quality of patients in both the intervention group and control group ameliorated after the intervention, and there were statistical differences compared with that before the intervention. The results of this meta-analysis showed that the sleep quality scores of patients in the intervention group [SMD = − 0.79, 95% CI (− 0.93, − 0.66), *p* < 0.01] were higher than those in the control group (Fig. 3), suggesting aromatherapy had a moderate effect on improving sleep quality. Sensitivity analysis showed that the analysis model was relatively stable, and the funnel plot demonstrated a moderate publication bias (Fig. 4).

### Subgroup and sensitivity analysis

#### Treatment status

Aromatherapy could significantly improve the sleep quality of patients undergoing chemotherapy [SMD = -0.94, 95%CI (− 1.29, − 0.58), *p* < 0.01], and it had moderate effects for patients in the perioperative period [SMD = -0.92, 95%CI (− 1.55, − 0.30), *p* < 0.01] (Table 2).

**Table 2:** Subgroup analyses of effect of aromatherapy on sleep quality

Subgroup	K	ES	Sample size		Random-effects analysis				Fixed-effects analysis			
					SMD	95%CI		*P*	SMD	95%CI		*P*
			EG	CG		L	U			L	U	
Treatment status												
Undergoing chemotherapy	3	5	176	164	-0.94	−1.29	−0.58	< 0.01	− 0.95	− 1.18	− 0.72	< 0.01
Perioperative period	4	4	161	155	− 0.92	− 1.55	− 0.30	< 0.01	− 0.89	− 1.13	− 0.66	< 0.01
Type of cancer												
Breast cancer	2	2	128	113	−0.98	−1.57	− 0.40	< 0.01	− 0.99	−1.27	−0.72	< 0.01
Digestive system cancers	2	2	94	95	−0.58	− 0.98	− 0.18	< 0.01	− 0.60	− 0.90	−0.31	< 0.01
Mixed types	3	5	157	150	−0.58	− 0.87	− 0.30	< 0.01	−0.58	− 0.81	−0.34	< 0.01
Control group												
Placebo	3	4	126	125	−0.64	−0.89	− 0.38	< 0.01	− 0.64	−0.89	− 0.38	< 0.01
Usual care	7	10	348	334	−0.88	−1.27	−0.49	< 0.01	− 0.86	− 1.02	− 0.70	< 0.01
Type of aroma oils												
Single essential oil	7	11	391	366	−0.94	−1.25	− 0.62	< 0.01	− 0.91	−1.07	− 0.76	< 0.01
Lavender	5	6	241	216	−1.06	−1.49	−0.63	< 0.01	− 1.05	− 1.25	− 0..85	< 0.01
others	4	5	150	150	−0.77	−1.22	− 0.31	< 0.01	− 0.72	− 0.96	− 0.48	< 0.01
Compound essential oil	2	2	61	65	−0.21	−0.57	0.14	0.23	−0.21	−0.57	0.14	0.23
Dosage of aroma oils (per time)												
2-4 drops	5	8	292	268	−0.78	−1.04	−0.51	< 0.01	− 0.77	− 0.95	−0.60	< 0.01
5-8 drops	3	4	95	101	−1.12	−2.06	0.19	0.02	−1.16	−1.47	−0.84	< 0.01
Frequency (per day)												
1 time	7	10	366	367	−0.86	−1.22	− 0.49	< 0.01	− 0.83	− 0.99	−0.68	< 0.01
> 1 time	3	4	108	92	−0.66	− 0.94	− 0.37	< 0.01	− 0.66	− 0.94	−0.37	< 0.01
Dimension of sleep												
Sleep latency	5	5	186	196	−0.78	−1.15	− 0.42	< 0.01	− 0.60	− 0.67	−0.52	< 0.01
Sleep duration	4	4	159	166	−0.17	− 0.58	0.24	0.43	−0.35	− 0.45	− 0.26	< 0.01
Sleep efficiency	3	3	137	138	−0.16	−0.64	0.33	0.53	−0.47	−0.54	− 0.04	< 0.01
Sleep disturbance	3	3	99	106	−0.58	−0.80	− 0.36	< 0.01	− 0.58	−0.80	0.36	< 0.01

*Note*: *K* number of studies, *ES* number of effect size, *AG* aromatherapy group, *CG* control group, *SMD* standardized mean difference effect size, *L* lower, *U* upper

#### Types of cancer

Aromatherapy could significantly ameliorate sleep quality in patients with breast cancer [SMD = -0.98, 95% CI (− 1.57, − 0.40), *p* < 0.01]. It had a moderate effect on patients with digestive system cancers [SMD = -0.58, 95%CI (− 0.98, − 0.18), p < 0.01] and mixed cancer types [SMD = -0.58, 95% CI (− 0.87, − 0.30), *p* < 0.01] (Table 2).

#### Control group

Both placebo [SMD = -0.64, 95%CI (− 0.89, − 0.38), *p* < 0.01] and usual care [SMD = -0.88, 95%CI (− 1.27, − 0. 49), *p* < 0.01] had an effect on sleep quality in cancer patients. There is no significant difference between them.

#### Types of aroma oils

Single essential oils had a significant effect on the sleep quality of cancer patients [SMD = -0.94, 95%CI (− 1.25, − 0.62), *p* < 0.01] while compound essential oils had no effect on sleep quality improvement [SMD = -0.21, 95%CI (− 0.57, − 0.14), *p* = 0.23]. In four included studies, single essential oils were subdivided into lavender essential oils and other single essential oils. It was shown that lavender essential oil and other single essential oils both had a significant effect on the sleep quality of cancer patients, but the effect of lavender essential oil was much better [SMD = -1.06, 95%CI (− 1.49, − 0.63), *p* < 0.01] (Table 2).

#### Dosage of aroma oils

In terms of the aroma oils drops used per time, 2-4 drops of essential oil had a moderate effect on sleep quality [SMD = -0.78,95%CI (− 1.04, − 0.51), *p* < 0.01] and 5-8 drops also had an effect [SMD = -1.12, 95%CI (− 2.06, 0.19), *p* = 0.02] (Table 2).

#### Frequency of intervention

Whether given once a day [SMD = -0.86, 95%CI (− 1.22, − 0.49), *p* < 0.01] or more than once a day [SMD = -0.66, 95%CI (− 0.94, − 0.37), *p* < 0.01], both frequencies of intervention had a moderate effect on improving sleep quality.

#### Dimensions of sleep

The dimensions of sleep include sleep latency, disturbance, efficiency and duration. Sleep latency refers to the time to fall asleep, from going to bed to actually falling asleep. Sleep disturbance refers to the clinical syndrome caused by the rhythm disorder of sleep awakening due to various reasons, resulting in abnormal sleep quality and abnormal behavior during sleep. Sleep efficiency refers to the actual sleep time divided by the time spent in bed between going to bed and waking up. Sleep duration refers to the average actual sleep hours per night in the past month. Aromatherapy had a moderate effect on the sleep latency of cancer patients [SMD = -0.78, 95%CI (− 1.15, − 0.42), *p* < 0.01], and it also had a moderate effect on sleep disturbance [SMD = -0.58, 95%CI (− 0.80, − 0.36), *p* < 0.01]. As for sleep efficiency, its effect was not statistically significant [SMD = -0.16, 95% CI (− 0.64, − 0.33), *p* = 0.53], Aromatherapy also did not affect sleep duration [SMD = − 0.17, 95% CI (− 0.58, 0.24), *P* = 0.43]. However, sensitivity analysis showed that Aromatherapy affected sleep efficiency and sleep duration, which means the model was unstable.

### Adverse events

Seven trials did not report the occurrence of adverse events while 3 trials did [16, 20, 21], of which 2 trials [16, 21] had no adverse events and only one trial [20] reported the adverse reactions were headaches and sneezing. However, no serious events were directly associated with aromatherapy (Table 1).

## Discussion

This meta-analysis including ten RCTs showed that aromatherapy interventions had a statistically moderate effect on improving the sleep quality of cancer patients, which is consistent with previous studies on the effects of aromatherapy on sleep quality [11, 25, 26]. Previous studies on aromatherapy have shown significant results in different groups of patients with sleep disorders, such as menopausal women [27], older patients with heart disease [28, 29], patients with burns [30], diabetes patients [31], patients with mental disorders [32], college students [33] and medical workers [34]. Due to the complexity of the patient population, the results cannot fully account for the effects of aromatherapy on cancer patients.

A meta-analysis of qualitative studies shows that patients with advanced cancer benefit from aromatherapy with positive effects on their physical symptoms and psychology, including reduced pain, increased ability to sleep, improved well-being, relief from stress and escape from illness [35]. Subgroup analysis indicated that the effect of aromatherapy on sleep quality was significant for patients receiving chemotherapy and in the perioperative period. Subgroup analysis of cancer types showed that aromatherapy intervention had a moderate effect on digestive system cancers and mixed cancer types while having a significant effect on improving sleep quality in breast cancer patients, which may be related to the fact that all breast cancer patients included were women. A qualitative study shows that aromatherapy massage seems to have physical and psychological benefits for women with cancer [36]. Due to their physiological and psychological characteristics, women are more sensitive to odor than men [37]. These results suggest that aromatherapy can be recommended as a complementary and alternative therapy in addition to drug therapy.

The exact mechanism of aromatherapy is not clear, but it is generally believed that essential oils can bind to nasal epithelial receptors and transmit neural signals to the brain, limbic system, and thalamus to balance and treat the individual mentally and physically through the release of endorphins and serotonins [38–42]. The type of essential oil may affect how well it binds to these receptors. Therefore, one important factor influencing the effect of aromatherapy is the type of plant essential oil. Lavender, peppermint, orange and chamomile are common in plant essential oils. Lavender is a member of the mint family and contains linalyl acetate, linalool and caryophyllene [43]. Orange essential oil, the main component of which is limonene, has a pleasant aroma, anti-anxiety and relaxation effects [44]. Chamomile essential oil is an aromatic ester that can relieve tension and anxiety and is calming and sleeping, and therefore has a strong psychotherapeutic healing effect [45]. The subgroup analysis of aromatic essential oils showed that single essential oils had a moderate effect on the sleep quality of cancer patients. Lavender essential oil was selected in five of the eight RCTs that used single essential oils, and it had a better effect on sleep quality than other single essential oils. Lavender is a commonly used herbal substance, and studies have reported that lavender had the lowest risk of poisoning and allergic reactions compared with other herbal substances [46]. The components of lavender essential oil are absorbed through the lungs and nasotracheal mucosa, sending signals to the olfactory system that stimulate the brain to secrete neurotransmitters γ- aminobutyric acid. γ-aminobutyric acid has sedative, anti-anxiety and mental relaxation effects, thereby regulating sleep disorders [47]. In addition, the results of this study indicated, compared with single essential oils, compound essential oils had no effect on sleep quality improvement. There are several possible reasons behind this. According to subgroup analysis, in Kawabata’s study [18], patients received only one aromatherapy intervention; the low frequency of the intervention may be one reason for the insignificant improvement in sleep quality. In Qi’ study [24], compound essential oils were composed of single essential oils (lavender, geranium, bergamot) in a ratio of 1:2:3. Although lavender, geranium and bergamot alone can improve sleep quality, the effect of compound essential oils are not as good as that of single essential oils due to the change in the proportion of various substances. The insignificant effect of compound essential oils on sleep quality improvement may also be related to the small sample size included in this meta-analysis (only 2 studies), and more studies are needed to explore its effect in the future.

Among ten RCTs included in this meta-analysis, all the studies chose to provide aromatherapy intervention for patients in the evening. Therefore, we suggest that aromatherapy be provided in the evening to achieve satisfactory results. By analyzing the effects of aromatherapy on different dimensions of sleep, we found that aromatherapy had a significant effect on reducing sleep latency in cancer patients, resulting in improved sleep quality. In addition, aromatherapy was also effective in the treatment of sleep disturbance. However, the effect of aromatherapy in regulating sleep duration and sleep efficiency is still unknown, and a large number of trials are needed in the future to determine its effect. In terms of the dosage of essential oils used per time, we found that 2-4 drops of essential oils and 5-8 drops of essential oils were both effective in improving sleep quality. From this, we infer that 2-8 drops might be the appropriate dosage for aromatherapy. In terms of the frequency of intervention, seven of the RCTs provided aromatherapy once a day while the other three gave it more than once a day. Subgroup analysis showed that both frequencies of intervention had a moderate effect on sleep quality improvement. Therefore, it can be concluded that aromatherapy is effective in improving sleep quality of cancer patients regardless of the frequency of intervention. Future studies with larger sample sizes are needed to verify our inference.

### Limitations of the current study

Although this meta-analysis provides a comprehensive review of the literature on improving sleep quality of cancer patients with aromatherapy, it still has some limitations. Firstly, the number of included trials was not very large, with only ten RCTs. Secondly, the quality of the included trials was not very high. Thirdly, due to the limitation of original data, there were not enough reports on the type and procedure of aromatherapy in the included trials, so we did not conduct a subgroup analysis of inhalation and massage aromatherapy. Fourth, due to the difference in duration of intervention in this study, we did not perform a subgroup analysis of the intervention duration. In addition, the minimum clinically important difference (MCID) values of the two scales used to measure sleep quality were uncertain. Therefore, in future meta-analyses, trials with a larger sample size and a multi-center design can be included to provide more reliable guidance for clinical sleep care of cancer patients.

## Conclusion

Aromatherapy can significantly improve the sleep quality of cancer patients and can be considered as a complementary therapy for sleep care in cancer patients. It is especially effective for breast cancer patients, patients undergoing chemotherapy, and those in the perioperative period. 2-8 drops are the proper dosage of essential oils used per time. The effect of single essential oils is better than that of compound essential oils, and lavender essential oils has the best effect, so it can be considered as a priority in treatment.

## Supplementary Information

**Additional file 1: Appendix.** A detailed search strategy for PubMed.

**Additional file 2: Supplement Table 1.** Description of assessment tools.

## Data Availability

Data supporting our findings are contained within the manuscript.

## Abbreviations

RCTs: randomized controlled trials
RCSQ: Richards-Campbell Sleep Questionnaire
PSQI: Pittsburgh Sleep Quality Index
MD: Mean difference
WMD: Weighted Mean difference
SMD: Standard Mean difference

## References

[1] Mogavero MP, DelRosso LM, Fanfulla F, Bruni O, Ferri R (2021). Sleep disorders and cancer: state of the art and future perspectives. Sleep Med Rev.

[2] Sharma N, Hansen CH, O'Connor M, Thekkumpurath P, Walker J, Kleiboer A (2012). Sleep problems in cancer patients: prevalence and association with distress and pain. Psycho-oncology.

[3] Savard J, Simard S, Blanchet J, Ivers H, Morin CM (2001). Prevalence, clinical characteristics, and risk factors for insomnia in the context of breast cancer. Sleep.

[4] Degner LF, Sloan JA (1995). Symptom distress in newly diagnosed ambulatory cancer patients and as a predictor of survival in lung cancer. J Pain Symptom Manag.

[5] Ford ES, Cunningham TJ, Giles WH, Croft JB (2015). Trends in insomnia and excessive daytime sleepiness among U.S. adults from 2002 to 2012. Sleep Med.

[6] Riemann D, Baglioni C, Bassetti C, Bjorvatn B, Dolenc Groselj L, Ellis JG (2017). European guideline for the diagnosis and treatment of insomnia. J Sleep Res.

[7] Dzierzewski JM, Dautovich N, Ravyts S (2018). Sleep and cognition in older adults. Sleep Med Clin.

[8] Tariq SH, Pulisetty S (2008). Pharmacotherapy for insomnia. Clin Geriatr Med.

[9] Posadzki P, Watson LK, Alotaibi A, Ernst E (2013). Prevalence of use of complementary and alternative medicine (CAM) by patients/consumers in the UK: systematic review of surveys. Clin Med (London, England).

[10] Lua PL, Zakaria NS (2012). A brief review of current scientific evidence involving aromatherapy use for nausea and vomiting. J Altern Complement Med (New York, NY).

[11] Hwang E, Shin S (2015). The effects of aromatherapy on sleep improvement: a systematic literature review and meta-analysis. J Altern Complement Med (New York, NY).

[12] Fradelos E, Komini A. The use of essential oils as a complementary treatment for anxiety. Am J Nurs Sci. 2014;(2). 10.11648/j.ajns.s.2015040201.11.

[13] Penny R (2011). Fundamentals of complementary and alternative medicine, 4th edition [book review]. J Australian Traditional-Med Soc.

[14] Moher D, Liberati A, Tetzlaff J, Altman DG, PRISMA Group (2009). Preferred reporting items for systematic reviews and meta-analyses: the PRISMA statement. PLoS Med.

[15] Feifeng Z, Wang Yan CHEN, Qingyue. (2021). Effect of aromatherapy on cancer-related fatigue and sleep quality in patients with hepatocellular carcinoma after Transarterial chemoembolization [J]. Chin Foreign Med Res.

[16] Shammas RL, Marks CE, Broadwater G, Le E, Glener AD, Sergesketter AR, et al. The effect of lavender oil on perioperative pain, anxiety, depression, and sleep after microvascular breast reconstruction: a prospective, single-blinded, randomized, controlled trial. J Reconstr Microsurg. 2021. 10.1055/s-0041-1724465 Advance online publication. 10.1055/s-0041-1724465 33548936

[17] Hamzeh S, Safari-Faramani R, Khatony A (2020). Effects of aromatherapy with lavender and peppermint essential oils on the sleep quality of cancer patients: a randomized controlled trial. Evid-based Complement Altern Med: eCAM.

[18] Kawabata N, Hata A, Aoki T (2020). Effect of aromatherapy massage on quality of sleep in the palliative care Ward: a randomized controlled trial. J Pain Symptom Manag.

[19] Li QY, Chen HL, Tian DX, Zhou JS, Zhao LL (2020). The effect of aromatherapy on insomnia and quality of life in breast cancer patients with perichemotherapy. Chin J Modern Nurs.

[20] Heydarirad G, Keyhanmehr AS, Mofid B, Nikfarjad H, Mosavat SH (2019). Efficacy of aromatherapy with Rosa damascena in the improvement of sleep quality of cancer patients: a randomized controlled clinical trial. Complement Ther Clin Pract.

[21] Blackburn L, Achor S, Allen B, Bauchmire N, Dunnington D, Klisovic RB (2017). The effect of aromatherapy on insomnia and other common symptoms among patients with acute leukemia. Oncol Nurs Forum.

[22] Ozkaraman A, Dügüm Ö, Özen Yılmaz H, Usta Yesilbalkan Ö (2018). Aromatherapy: the effect of lavender on anxiety and sleep quality in patients treated with chemotherapy. Clin J Oncol Nurs.

[23] Li J, Qin W, Liu F (2018). The effect of aromatherapy on perioperative anxiety and sleep quality in patients with gastric cancer. Chin J Modern Nursing.

[24] Qi HY, Yang JL, Wang CX, Li LS (2016). Effects of aural acupoint pressure on sleep quality in perioperative patients with colorectal cancer. Western Chin Med.

[25] Kim ME, Jun JH, Hur MH (2019). J Korean Acad Nurs.

[26] Lin PC, Lee PH, Tseng SJ, Lin YM, Chen SR, Hou WH (2019). Effects of aromatherapy on sleep quality: a systematic review and meta-analysis. Complement Ther Med.

[27] Dos Reis Lucena L, Dos Santos-Junior JG, Tufik S, Hachul H (2021). Lavender essential oil on postmenopausal women with insomnia: double-blind randomized trial. Complement Ther Med.

[28] Cheraghbeigi N, Modarresi M, Rezaei M, Khatony A (2019). Comparing the effects of massage and aromatherapy massage with lavender oil on sleep quality of cardiac patients: a randomized controlled trial. Complement Ther Clin Pract.

[29] Jodaki K, Abdi K, Mousavi MS, Mokhtari R, Asayesh H, Vandali V (2021). Effect of rosa damascene aromatherapy on anxiety and sleep quality in cardiac patients: a randomized controlled trial. Complement Ther Clin Pract.

[30] Rafii F, Ameri F, Haghani H, Ghobadi A (2020). The effect of aromatherapy massage with lavender and chamomile oil on anxiety and sleep quality of patients with burns. Burns.

[31] Nasiri Lari Z, Hajimonfarednejad M, Riasatian M, Abolhassanzadeh Z, Iraji A, Vojoud M (2020). Efficacy of inhaled Lavandula angustifolia mill. Essential oil on sleep quality, quality of life and metabolic control in patients with diabetes mellitus type II and insomnia. J Ethnopharmacol.

[32] Zhou AH, Zeng XL, Mo XX (2020). Influence of lavender aromatherapy on negative emotion and sleep quality in female patients with remitted schizophrenia. Guangxi Med J.

[33] Yang QY, Hou JJ (2020). The influence of TCM aromatherapy and psychological intervention on insomnia in undergraduates. Wester J Trad Chin Med.

[34] Liu XX, Liu YT, Sun HM (2021). Effect of traditional Chinese medicine combined with inhalation aromatherapy on insomnia of Junior nurses. J Liaoning Univ Tradit Chin Med.

[35] Armstrong M, Flemming K, Kupeli N, Stone P, Wilkinson S, Candy B (2019). Aromatherapy, massage and reflexology: a systematic review and thematic synthesis of the perspectives from people with palliative care needs. Palliat Med.

[36] Ho S, Kwong A, Wan K, Ho R, Chow KM (2017). Experiences of aromatherapy massage among adult female cancer patients: a qualitative study. J Clin Nurs.

[37] Xiao Y, Li L, Xie Y, Xu J, Liu Y (2018). Zhong nan da xue xue bao. Yi xue ban = Journal of Central South University Medical sciences.

[38] Lane B, Cannella K, Bowen C, Copelan D, Nteff G, Barnes K (2012). Examination of the effectiveness of peppermint aromatherapy on nausea in women post C-section. J Holistic Nurs.

[39] Stringer J, Donald G (2011). Aromasticks in cancer care: an innovation not to be sniffed at. Complement Ther Clin Pract.

[40] Marzouk TM, El-Nemer AM, Baraka HN (2013). The effect of aromatherapy abdominal massage on alleviating menstrual pain in nursing students: a prospective randomized cross-over study. Evid-based Complement Altern Med: eCAM.

[41] Tournaire M (2004). Alternative approaches to pain relief during labor and delivery. Adv Exp Med Biol.

[42] Smith CA, Collins CT, Crowther CA. Aromatherapy for pain management in labour. Cochr Database Syst Rev. 2011;(7):CD009215. 10.1002/14651858.CD009215. 10.1002/14651858.CD009215 PMC12333927 21735438

[43] Keene MR, Heslop IM, Sabesan SS (2019). Complementary and alternative medicine use in cancer: a systematic review. Complement Ther Clin Pract.

[44] Zhang N, Hu YX (2013). Anxiolytic efficacy of 4 kinds of essential oil. J Shanghai Jiao Tong Univ (Agricultural Science).

[45] Li H, Lin L, Li M, Xiong P, Tang P (2016). Efficacy of aromatherapy in improving elderly’s sleep quality. J Chengdu Med College.

[46] Koulivand PH, Khaleghi Ghadiri M, Gorji A (2013). Lavender and the nervous system. Evid-based Complement Altern Med: eCAM.

[47] LQ, R M, Y MF (2020). Efficacy of aromatherapy in improving sleep quality and nausea and vomiting in patients with concurrent Chemoradiotherapy for cervical cancer. Med Innov China.

