# Supplementary material for: Aromatherapy with single essential oils can significantly improve the sleep quality of cancer patients: a meta-analysis
Source: BMC Complement Med Ther. 2022 Jul 14;22:187. doi: 10.1186/s12906-022-03668-0 (PMC9284915; doi:10.1186/s12906-022-03668-0)
Supplement: Supplementary file 1 — Additional file 1: Appendix. A detailed search strategy for PubMed. [file 12906_2022_3668_MOESM1_ESM.docx]

**Appendix. A detailed search strategy for PubMed**

#1 "Neoplasms"[MeSH Terms] OR "Neoplasia"[Title/Abstract] OR "Neoplasias" [Title/ Abstract] OR "Neoplasm"[Title/Abstract] OR "Tumors"[Title/Abstract] OR Tumor" [Title /Abstract] OR "Cancer"[Title/Abstract] OR "Cancers"[Title/Abstract] OR "Malignancy" [Title/Abstract] OR "Malignancies"[Title/Abstract] OR "malignant neoplasms" [Title/Abstract] OR "malignant neoplasm"[Title/Abstract] OR "neoplasm malignant" [Title/ Abstract] OR "neoplasms malignant"[Title/Abstract] OR "benign neoplasms"[Title/ Abstract] OR "neoplasms benign"[Title/Abstract] OR "benign neoplasm" [Title/Abstract] OR "neoplasm benign"[Title/Abstract]

#2 "Sleep Wake Disorder"[Mesh] OR "Sleep"[Mesh] OR "Wakefulness"[Mesh] OR "Dyssomnias"[Mesh] OR "Sleep Disorders, Intrinsic"[Mesh] OR "Sleep Initiation and Maintenance Disorders"[Mesh] OR "insomnia*"[Title/Abstract] OR "dyssomnia*" [Title/Abstract] OR "sleep*"[Title/Abstract] OR " wakeful*"[Title/Abstract]

#3 "Aromatherapy"[Mesh] OR "Oils,Volatile"[Mesh] OR "Lavandula*"[Mesh] OR "Plant Oils "[Mesh] OR "Smell "[Mesh] OR "Aromatherapies"[Title/Abstract] OR "Aroma Therapies"[Title/Abstract] OR "Aroma Therapy"[Title/Abstract] OR "Therapy, Aroma" [Title/Abstract] OR "Therapies, Aroma "[Title/Abstract] OR "essential oils"[Title/Abstract] OR "Oils, Plant"[Title/Abstract] OR " Plant Oil"[Title/Abstract] OR "Oil, Plant" [Title/Abstract] OR " Vegetable Oils"[Title/Abstract] OR "Oil, Vegetable"[Title/Abstract] OR "Sense of Smell"[Title/Abstract] OR "Volatile Oils"[Title/Abstract] OR "Oil, Essential"[Title/Abstract] OR "Essential Oil"[Title/Abstract] OR "Oils, Essential" [Title/Abstract] OR "Oils, Essential"[Title/Abstract] OR "Essential Oils"[Title/Abstract] OR "Volatile Oil"[Title/Abstract] OR "Oil, Volatile"[Title/Abstract] OR "La vender"[Title/Abstract] OR "Lavenders"[Title/Abstract] OR "Lavandula angustifolia" [Title/Abstract] OR "Uses, Therapeutic"[Title/Abstract] OR "Therapeutic Use" [Title/Abstract] OR "Therapeutic Effects"[Title/Abstract] OR "Oil, Volatile"

[Title/Abstract] OR "Therapeutic Effect"[Title/Abstract] OR "Effect, Therapeutic"

[Title/Abstract] OR " Use, Therapeutic"[Title/Abstract] OR " Effects, Therapeutic" [Title/Abstract]

#4 "Randomized Controlled Trial"[Publication Type] OR "randomized"[Title/Abstract] OR "placebo"[Title/Abstract] OR " double-blind"[Title/Abstract]

#5 #1AND #2 AND #3 AND #4
