# Supplementary material for: Aromatherapy with single essential oils can significantly improve the sleep quality of cancer patients: a meta-analysis
Source: BMC Complement Med Ther. 2022 Jul 14;22:187. doi: 10.1186/s12906-022-03668-0 (PMC9284915; doi:10.1186/s12906-022-03668-0)
Supplement: Supplementary file 2 — Additional file 2: Supplement Table 1. Description of assessment tools. [file 12906_2022_3668_MOESM2_ESM.doc]

| **Supplement Table 1** Description of assessment tools | | |
| --- | --- | --- |
| No. | Type of assessment tools | Description of assessment tools |
| 1 | The Richards-Campbell Sleep Questionnaire (RCSQ) | RCSQ is a five-item, visual analogue scale which was designed as an outcome measure for assessing the perception of sleep in critically ill patients. The scale evaluates perceptions of depth of sleep, sleep onset latency, number of awakenings, time spent awake, and overall sleep quality, with high internal consistency (Cronbach’s α = 0.90). Each question in RCSQ has a maximum score of 100, with higher scores meaning better sleep quality. |
| 2 | The Pittsburgh Sleep Quality Index (PSQI) | PSQI is a self-rated questionnaire for the assessment of sleep quality and widely applicable to the general population. PSQI consists of seven dimensions: subjective sleep quality, sleep latency, sleep duration, habitual sleep efficiency, sleep disturbances, use of sleeping medication, and daytime dysfunction, with good internal consistency (Cronbach’s α = 0.78). Scores on each of the seven PSQI subscales are used to calculate a global score that ranges from 0 to 21. The lower the global score, the better the sleep quality, with a global score ≤ 5 indicating good sleep quality. |
